# Biomaterials Approaches to Combating Oral Biofilms and Dental Disease

**DOI:** 10.1186/1472-6831-6-S1-S15

**Published:** 2006-06-15

**Authors:** James D Bryers, Buddy D Ratner

**Affiliations:** 1 Department of Bioengineering, Biomaterials (UWEB) Center, University of Washington, Seattle, WA 98195, USA; 2 University of Washington Engineered Biomaterials (UWEB) Center, University of Washington, Seattle, WA 98195, USA

## Abstract

**Background:**

Possibilities for biomaterials to impact the dental caries epidemic are reviewed with emphasis placed on novel delivery biomaterials and new therapeutic targets.

## Introduction

### The Problem

Although significant progress has been made in reducing and controlling dental caries, the disease still remains a problem for many children and adults in the United States. The problem is much worse for children from poor families. More than a third of all children 2 to 9 years old living in poverty in the U.S. have one or more untreated primary teeth that are affected with caries. There are significant and persistent racial disparities in dental health and treatment.

Dental caries is an infectious, communicable disease resulting in destruction of tooth structure by acid-forming bacteria found in dental plaque, an intraoral biofilm. The disease results in tooth loss if left untreated. Fluoride in the mouth can inhibit demineralization in early carious lesions and promote remineralization – leading to rebuilt and stronger outer layers of the tooth (*i.e*., enamel on the crown of the tooth and cementum on the root). Water fluoridation, use of other fluoride therapies such as daily tooth brushing with fluoride-containing toothpaste, placement of dental sealants and fluoride-releasing filling materials, and other appropriate oral health care approaches currently are utilized in the prevention and control of dental caries. The mainstays of caries prevention, topical and systemic fluorides and pit and fissure sealants, are technologies developed in the 1950s and 1960s. Nevertheless, the relapse rates for children who have been treated for advanced caries are very high.

Science and technology have not produced sufficient practical tools for public health practitioners and the private delivery system to address the pandemic in dental caries that exists for children and adults from families with low incomes and for numerous ethnic and racial minority groups. Moreover, it is unclear whether the barriers are remediable bioengineering and technical problems or fundamental science questions. Nevertheless, the obligation to address the gap between scientific research and practical application is especially relevant today. The federal government and state governments bear the majority of the cost of trying to control this pandemic through Medicaid, the Public Health Service, Indian Health Service, and other public programs. These costs continue to escalate, and continued applications of existing technology are unlikely to reduce these disparities.

This paper reviews emerging biomaterials technologies that may be translated into strategies for more effective means of controlling oral biofilms, dental caries, and periodontal disease.

### Processes Governing Biofilm Formation

Microbial cells (predominantly bacteria) in an extracellular polymer substratum are called *biofilms *[1]. Complex physical, chemical, and biological processes govern bacterial attachment, biofilm formation, and persistence [1]. The following fundamental processes comprise the development of a bacterial biofilm: (1) Substratum pre-conditioning by circumstantial or pre-meditated adsorption of fluid phase organic molecules; (2) bacterial cell transport to the surface; (3) cell desorption from the substratum; (4) permanent cell adhesion to the substratum; (5) bacterial metabolism (cell substrate conversion; cell growth and replication; extracellular exopolymer production; cell starvation, death, lysis); and (6) biofilm removal (cell and biofilm detachment; biofilm sloughing). Naturally, the relative influence of each process is dependent upon the specific system, the prevailing environmental conditions, and biological changes throughout the lifetime of the biofilm.

A diverse community of bacteria comprises tooth plaque biofilm. Early tooth colonizers (*e.g., Streptococcus, Hemophilus, Neisseria*, and *Veillonella*) adhere to enamel pellicle protein layers via specific and non-specific adhesion mechanisms. Subsequently, other species adhere to the developing biofilm. Once established, the biofilm flora remain relatively stable, despite periodic perturbations in the environment. Oral microbial disease is associated with major shifts in the biofilm ecology. Dental caries is associated with an increased frequency of consumption of fermentable sugars in the diet, which results in an increase in the acidogenic and aciduric species (*e.g., S. mutans, Lactobaccilli*). Factors affecting dental plaque include fermentable sugars, low pH, redox potential, and ambient nutrients.

### Emerging Approaches to Dental Caries Prevention

In the last 30 years a number of community- and individual-level strategies for preventing caries, notably water fluoridation and fluoridated toothpastes, have been highly successful. In light of fluoridation's success, a number of interventions have arisen for primary prevention of dental caries in individual at-risk patient groups. Regretably, these interventions have existed for some time and are still inadequate to resolve the caries pandemic in disparity populations. These existing interventions include: application of acidulated phosphate fluoride gel (APF), fluoride varnish, chlorhexidine varnishes and gels (not available in the United States), pit and fissure sealants, and the use of dentrifices and other products containing antimicrobials (*e.g*., triclosin and sanguinaria), noncariogenic sweeteners (*e.g*., xylitol), or agents to promote remineralization (*e.g*., Enamelon toothpaste). Fluoride reservoir systems, developed for post radiation cancer patients, successfully controlled caries but were never brought to market.

A number of the key processes controlling biofilm formation provide targets for application of novel preventive or remedial technologies (Table 1). Given the increasing use of relatively invasive medical and surgical procedures, the material properties of medical devices have received much attention, as have strategies to target antimicrobials to prevent device-related infections. Dental plaque and oral hygiene would appear to be obvious therapeutic targets for the application of novel anti-infective strategies.

Prospects include: *(i) *materials or surface coatings that prevent bacterial adhesion, *(ii) *surfaces that phase change upon command, and *(iii) *passive or active controlled release of anti-infective agents.

### Materials or Surface Coatings That Prevent Bacterial Adhesion

When applied to surfaces or incorporated directly within the biomaterial, poly(ethylene oxide) (PEO) can inhibit protein adsorption and bacterial adhesion. This polymer and its derivatives can be very effective in reducing adhesion from 60–90% for a variety of bacterial species.

For example, we have developed PEO-like oligoglyme coatings [CH_3_-O-(CH_2_-CH_2_-O) *n*-CH_3_, in which *n *= 1–4], using radio frequency gas plasma discharge techniques that can apply these coating to a variety of base materials [2]. The degree to which this family of coatings protects surfaces depends in part on which oligomer is used and what bacterial species is being used. In our studies with *Pseudomonas aeruginosa*, a tetraglyme (*n *= 4) coating can reduce colonization by 99% relative to uncoated controls. In other experiments, tetraglyme was deposited on glass in a pattern using photolithographic methods before exposure to a suspension of *Methylobacterium extorquens*. After 72 hours, almost all of the bacteria were adhering to the bare glass, with very few bacteria on the glyme-coated regions.

The elegance of the RF plasma technique is that such PEG-like coatings can be deposited onto a variety of existing materials and geometric shapes (*e.g*., inside and outside of catheters, porous scaffold structures, metals, polymers, ceramics, orthodontic materials, and enamel). Although such glyme-plasma coatings resist protein adsorption for at least one week, longer-term bacterial challenges are needed to more fully evaluate these coatings.

### Surfaces That Change Phase on Command

The surface-grafted thermally responsive polymer poly(N-isopropyl acrylamide) (PNIPAM) has a critical-solubility temperature of about 32°C, making it insoluble in water at temperatures above 32°C but soluble at temperatures below 32°C.

In lab tests, >90% of microorganisms (*Staphylococcus epidermidis*, *Halomonas marina*) and naturally-occurring marine microorganisms that attached to PNIPAM grafted to polystyrene surfaces were removed immediately when the hydration state of the polymer was changed by simply rinsing the samples in cold (4°C) water [3]. Confluent sheets of mammalian cells can also be rapidly detached from similar PNIPAM-grafted surfaces when the temperature is shifted from 37°C to room temperature. Whether temperature changes can remove thick bacterial biofilms from such surfaces remains to be evaluated.

### Passive or Active Controlled Release of Anti-infective Agents

There are numerous biomaterials platforms that have been used for decades for the controlled delivery of drugs including cancer chemotherapies, birth control, insulin, anti-viral agents, analgesics, antibiotics, and cosmetics.

Journals such as the *Journal of Biomedical Materials Research*, *Biomaterials*, and *Journal of Controlled Release *have been filled for decades with the scientific advances in controlled-therapy release. Many of these publications deal with oral biomaterials and oral hygiene but have unfortunately escaped the attention of most dental professionals.

Regrettably, due to page limits, this paper cannot possibly review even a fraction of the drug delivery systems available. The reader is directed to several reviews on this topic [4-9]. Here we will discuss the various types of controlled-release systems available (passive versus controlled or activated) and enumerate the possible agents that could be released to control dental caries.

### Passive Delivery Systems

A convenient classification of controlled-release systems is based on the mechanism that controls the release of the substance in question. The most common mechanism is diffusion. Two types of diffusion-controlled systems have been developed; the first is a reservoir device in which the bioactive agent (drug) forms a core surrounded by an inert diffusion barrier. These systems include membranes, capsules, microcapsules, liposomes, and hollow fibers. The second type is a monolithic device in which the active agent is dispersed or dissolved in an inert polymer. As in reservoir systems, drug diffusion through the polymer matrix is the rate-limiting step, and release rates are determined by the choice of polymer and its consequent effect on the diffusion and partition coefficient of the drug to be released.

In chemically controlled systems, chemical control can be achieved using bioerodible or pendant chains. The rationale for using bioerodible (or biodegradable) systems is that the bioerodible devices are eventually absorbed by the body and thus need not be removed surgically. *Bioerosion *can be defined as the conversion of a material that is insoluble in water into one that is water-soluble. In bioerodible systems the drug is ideally distributed uniformly throughout a polymer in the same way as in monolithic systems. As the polymer surrounding the drug is eroded, the drug escapes. In a pendant chain system, the drug is covalently bound to the polymer and is released by bond scission, either hydrolytically or enzymatically. In solvent-activated controlled systems, the active agent is dissolved or dispersed within a polymeric matrix and is not able to diffuse through that matrix. As the environmental fluid (*e.g*. water) penetrates the matrix, the polymer swells and its glass transition temperature is lowered below the environmental (host) temperature. Thus, the swollen polymer is in a rubbery state and allows the drug contained within to diffuse through the encapsulant.

### Responsive Drug Delivery Systems

Responsive drug-delivery systems can be classified as open- or closed-loop systems. Open-loop systems are also called pulse or externally regulated systems; the rate of drug released is not dependent upon environmental conditions. The rate of drug released can be controlled and enhanced using external stimulants, such as magnetism and ultrasound. In *magnetically-controlled drug delivery devices*, small magnetic spheres are embedded in a drug-containing polymer. These spheres release a significant amount of drug when exposed to an oscillating field. Similarly, the release rate also increases when analogous drug-containing polymers are exposed to ultrasound. Ultrasound was found to enhance erosion and degradation of some biodegradable polymers [10] It also can act as an on-off switch as in certain drug delivery systems being developed in the University of Washington Engineered Biomaterials (UWEB) Center [11,12].

In closed-loop systems, or self-regulated systems, the release is in direct response to the conditions detected, be it temperature, type of solvent, pH, or concentration, to name a few. Poly(N-isopropylacrylamide) is a well-known example of a *thermo-responsive polymer*. At its transition of 32°C, the polymer is soluble in water; but, as temperature is increased, the polymer precipitates and phase separates. Poly(ethylene glycol) and poly(propylene glycol) copolymers as well as poly(lactic acid) and poly(glycolic acid) copolymers also exhibit thermo-responsiveness. These polymers are useful in developing thermogelling systems (*e.g*., Atridox^®^), in which the drug is initially dissolved in the liquid form of the polymer at room temperature. When this mixture is injected into the body at 37°C, the polymer turns into a gel, which eventually degrades and releases the drug molecules.

Self-regulating insulin-delivery devices depend on the concentration of glucose in the blood to control the release of insulin. One proposed system would immobilize glucose oxidase (an enzyme) to a pH-responsive polymeric hydrogel, which encloses a saturated insulin solution. At high glucose levels, glucose is catalyzed by glucose oxidase which converts it to gluconic acid, thus lowering the pH. This decrease in pH would cause the membrane to swell, forcing the insulin out of the device [13,14]. A similar pH responsive material could deliver "on-demand" anti-caries therapy at the first drop in pH.

### Anti-caries Therapeutics for Controlled Release

The chemical properties (hydrophobic vs. hydrophilic) of therapeutic agents selected for release will determine to some extent the possible controlled release system selected [15]. Numerous types of agents could be incorporated in a new generation of biomaterials for the prevention of dental caries. For the sake of brevity, these agents are simply listed with their mode of action in Table 2.

An example of such a system developed to eradicate biofilms involves liposomal delivery. Jones and colleagues have reported extensive studies of the interaction between liposomes and bacterial biofilms. Interestingly, when targeting mixed biofilms of *Streptococcus sanguis *and *S. salivarius *with liposomes loaded with the bactericide triclosan, anionic liposomes were most effective against *S. sanguis *but relatively ineffective against *S. salivarius *[16]. An additional approach has been to load antibacterials into liposomes adsorbed on the surface of zinc citrate particles, as used in toothpaste formulations, to produce solid supported vesicles containing either triclosan or aqueous-soluble penicillin-G. Other oral hygiene approaches have included liposomal encapsulation of the enzyme glucose oxidase and horseradish peroxidase. This process generates hydrogen peroxide and oxyacids in the presence of their substrates. These liposome systems were effective against *S. gordonii *biofilms in a manner dependent upon liposome-biofilm and substrate-biofilm incubation times [17].

## Conclusion

Increasing scientific research over the past 10 years in biofilm formation, especially in the oral cavity, has provided a wealth of possible targets with which to eradicate dental caries. Advances in the understanding of biofilm formation, coupled with emerging engineered biomaterials, provides many potential platforms and strategies that may be applied to prevent dental caries in susceptible populations.

## Competing interests

The author(s) declare that they have no competing interests.

## Authors' contributions

All authors read and approved the final manuscript.

## Figures and Tables

**Table 1:** Potential strategies to reduce or eliminate bacterial biofilms

Process contributing to Net Biofilm Accumulation	Potential Interventions
Substratum pre-conditioning	Change surface chemistry; non-fouling surfaces vs. signal decorated surfaces
Cell Deposition	Maintain sterility
Reversible adhesion Irreversible adhesion Desorption	Change surface chemistry or surface topography. Tethered or released toxic agent(s). Base material degradable. External toxic challenge. Specific adhesin blockers
Cell Signaling	Signal analogs; receptor blocking.
Cell Metabolism	Antibiotic challenge; starvation, chelator challenge; bacteriophage infection, bacteriophage lytic enzymes, device surgical removal
Biofilm Removal	Antibiotic challenge; starvation, chelator challenge; bacteriophage infection, bacteriophage lytic enzymes, device surgical removal

**Table 2:** Anti-caries Therapeutics for Controlled Release

Agent Released	Mode of Action	Relative Costs	Reference
Fluoride	mixed mode	low	Aaltonen *et al*., 2000 [18]
Chlorhexidine	disinfectant	low	Twetman, 2004 [19]
Xylitol	noncariogenic sweeteners	low	Smits & Arends, 1985. [20] Tanzer, 1995 [21]
Commercial antibiotics	mixed targets	moderate	
Anti-bacterial peptides (defensins, magainin derivatives, bacteriocins and cecropins)	cell membrane perforation	moderate/high	Otvos *et al*., 2004 [22] Ericksen *et al*., 2005 [23]
pH Buffers	maintain non-acidic pH	low	Lynch, 2004 [24]
Chelators (EGTA)	strips Ca^+2 ^from biofilm polysaccharide; causes biofilm disruption	low	Ozerdem *et al*., 2003 [25] Raad *et al*., 2003 [26]
Polysaccharide modifiers (lyase, AlCl_3_)		moderate	Albrecht and Schiller, 2005 [27] Stoodley *et al*., 2001 [28]
Enzymes (proteases)	enzymatically degrades cell walls and adhesin proteins	moderate/high	Berg *et al*., 2001 [29] Johansen *et al*., 1997 [30] Sato *et al*., 1983 [31]
Quorum sensing analogs (furanones, salicylic acid)	negates *las *and *Rhl *regulon systems, down-regulates polysaccharide synthesis	moderate/high	Daniels *et al*., 2004 [32] Baveja *et al*., 2004 [33]
Anti-adhesin blockers	negates specific adhesion of select bacteria to exclusive ligand	high	Hajishengallis *et al*., 1992 [34]
